# Vesicocolonic Fistula: A Sequela of Missed Blunt Abdominal Injury

**DOI:** 10.1155/2013/763032

**Published:** 2013-01-29

**Authors:** Abdulwahab Akanbi Ajape, AbdulLateef Babata, Mustapha Mohammed Kura, Musa Nuhu

**Affiliations:** ^1^Division of Urology, Department of Surgery, University of Ilorin and University of Ilorin Teaching Hospital, Nigeria; ^2^Department of Surgery, Federal Medical Centre, Gombe, Nigeria

## Abstract

A case of vesicocolonic fistula secondary to a missed abdominal injury is described. The patient, a 25-year-old male Hausa, Nigerian, was involved in a road traffic accident and sustained head injury with a fractured left femur and a missed blunt abdominal injury. He was referred to us about six months following the injury, on account of failure to micturate, recurrent passage of frequent watery stool, and recurrent fever and weight loss. A suspected diagnosis of intestinovesical fistula was confirmed on micturating cystography. He had a single stage repair of the vesico-colonic fistula. The repair of urethrocutaneous fistula was scheduled for a later date, which he later had. He was discharged but continued with orthopaedic consultation and management.

## 1. Introduction

Fistula formation between the bowel and the lower urinary tract is not frequently encountered [1]. Varying aetiological factors, most commonly from colonic diverticulitis, has been adduced for its occurrence. Congenital communication between the bowel and the urinary tract is seen with developmental anomalies of the anorectum and usually these manifest early in life. Acquired fistula formation between the bowel and the urinary tract has been documented to occur as a result of inflammatory bowel diseases and malignant diseases; both solid and haematologic malignancies [2–4]. And recently such fistula has been described in patient with AIDS [5]. 

The places of trauma in the aetiology of this fistula are mainly from iatrogenic trauma or from penetrating abdominal trauma. Vesicocolonic fistula (VCF) from blunt abdominal trauma is extremely rare and this warranted the documentation of this case.

## 2. Case Report

A 25-year-old Hausa apprentice boy was referred to us on account of failure to micturate and a discharging wound on the right side of the root of the penis. He was knocked off the road about six months prior to presentation, by a fast moving vehicle while riding bicycle on the highway. There was no known premorbid condition in him. He lost consciousness immediately and also sustained closed fracture of the left femur. He had initial resuscitation at the referring centre, where urethral catheter was passed among other things. The relation gave history of blood stained urine after the initial catheterization. He was on admission for about twelve weeks before he regained full consciousness. He was discharged against medical advice from the centre because he wanted to manage the bone fracture locally by the traditional bone setter.

He noticed difficulty with micturition soon after the urethral catheter was removed; this was characterised by occasional straining and passage of scanty urine. This necessitated presenting again in the hospital where he was recatheterised but no urine came through the catheter. At this time, he also noticed to have persistent and frequent passage of watery stool, appearance of boil at the root of the penis which later burst. There was associated fever with chills and he was unable to walk with the left lower limb.

**Figure 1:**
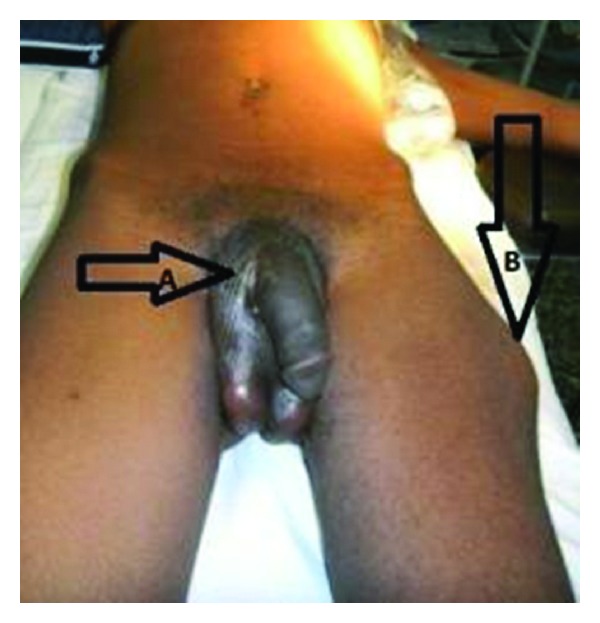
The urethrocutaneous fistula (horizontal arrow A) and the malalignment of the fractured left femur (vertical arrow B; facing downwards) are shown.

 At presentation to us, he was looking toxic, pale, and dehydrated with deformity of the left thigh the temperature was 38°C, pulse was 94 per minute, blood pressure was 110/60 mmHg and the chest was clinically clear, no murmur or gallop rhythm. There was right renal angle tenderness; the rectum was empty with no organomegaly. The urethral catheter was not functioning; a suspected urethrocutaneous fistula was noted on the right side of the root of the penis and a neglected malaligned fracture of the left femur (Figure 1).

A clinical diagnosis of vesicointestinal and urethrocutaneous fistulae with urosepsis in a patient with malunion of the left femoral fracture was made.

The admitting packed cell volume was 20% with leucocytosis of 20.6 × 10^9^/L and neutrophil of 75%. The platelet was adequate. The blood biochemistry revealed urea of 16 mmol/L but other parameters were within normal limits. Urinalysis and urine culture could not be done for lack of sample. The blood culture yielded no bacteria growth after 48-hour incubation. The abdominopelvic ultrasound showed mild fullness of the collecting system of the right kidney and nonvisualisation of the bladder.

He was optimised by correction of the fluid deficit. He had a combination of parenteral antibiotic therapy (ciprofloxacin, metronidazole, and gentamicin)and three pints of blood transfused.

**Figure 2:**
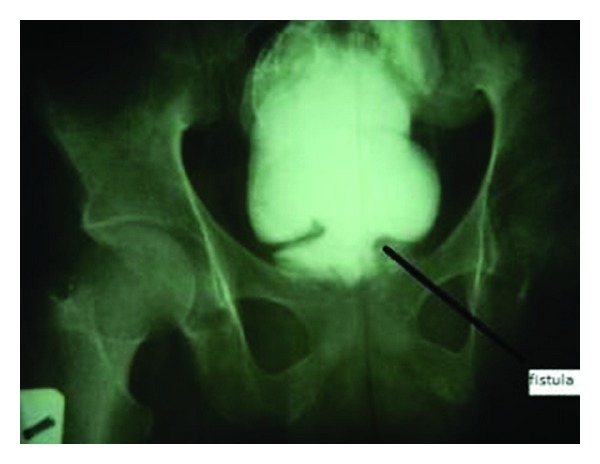
Micturating cystourethrogram radiograph showing contrast intravasation into the sigmoid colon during the filling phase of the cystography. Black arrow pointing at the vesicocolonic junction.

He subsequently had micturating cystourethrography which confirmed the fistulous connection with the sigmoid colon (Figure 2).

**Figure 3:**
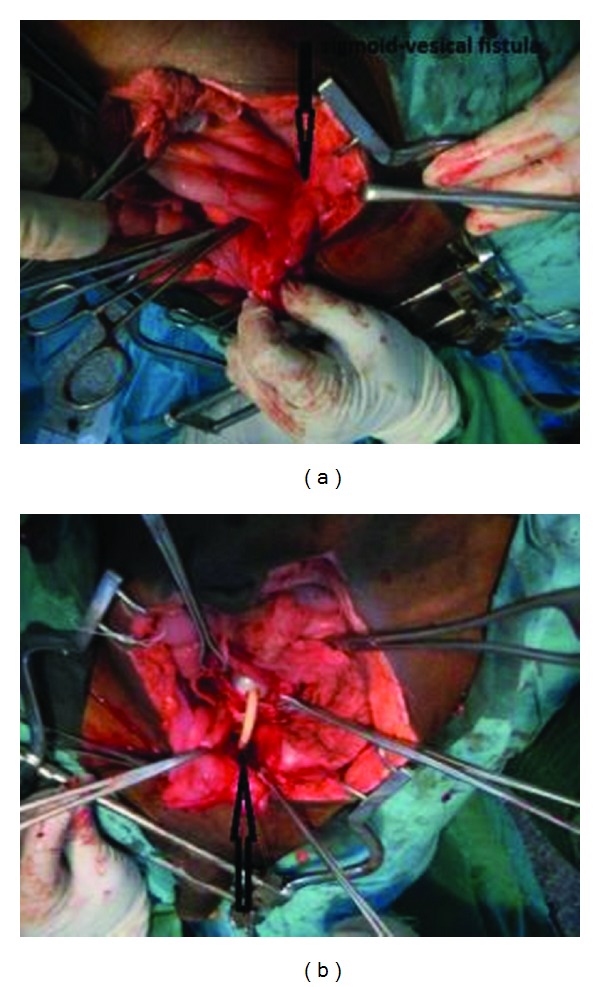
(a) Intraoperative photograph showing the sigmoid/vesical junction (arrow) before it was brought down. (b) Intraoperative photograph showing the bladder (arrow) with a Foley catheter going into the sigmoid colon (catheter balloon shown).

He consented for exploratory laparotomy and closure of the fistula which he had as a single stage procedure, without colostomy, after an initial bowel preparation. The intraoperative findings were those of fistulous connection with a diameter of 3.5 cm, between the fundus of the bladder and antimesenteric border of the sigmoid colon and minimal inflammatory adhesions in the pelvis. There was no evidence of colonic diverticulitis (Figures 3(a) and 3(b)). The fistulous connection was incised and the edges excised and freshened; the bladder was closed in two layers and extraperitonealised while the sigmoid was closed in single layer with interrupted vicryl 2/0 suture. The two ureters were stented with paediatric feeding tube (size 8) and brought out through a separate stab wound. A suprapubic cystostomy tube was left in place to protect the urethrocutaneous fistula. He did well postoperatively except for the superficial wound infection that he developed. The loose stool stopped and the cystostomy tube was draining urine before discharge. He later had repair of the urethrocutaneous fistula, which healed well. He was discharged for orthopaedic followup at the outpatient clinic.

## 3. Discussion

Trauma is emerging as a leading cause of death and significant morbidity all over the world, the developing nations inclusive. This has been attributed to increase technological activity. especially with the development of high speed automobiles; in addition to this, the poor states of our roads often contribute to this menace [6].

Report of VCF has been scanty in our setting principally because of the rarity of colonic diverticulitis and Crohn's disease [7]. While the pathophysiologic basis of VCF from iatrogenic or penetrating trauma may be easy to understand, that from blunt abdominal injury, as occurred in the present case, may not be as straight forward. In the absence of history or clinical findings on the patient to suggest penetrating injury to the abdomen and absence of pelvic fracture on the X-ray (Figure 2), it is imperative that blunt abdominal injury could have been missed. Although, enterovesical fistula has been documented as a rare complication of urethral catheterization in an irradiated bladder for urothelial tumour with prolonged urethral catheterization of about twelve weeks [8]. The aforementioned explanation may not adequately explain the present scenario. However, the possibility of a concomitant bladder and colonic rupture that was missed because the patient was unconscious may explain the genesis of the VCF. Another explanation may be intussusception of the sigmoid colon into an unsuspected bladder rupture which became entrapped and pressure necrosis of the sigmoid colon from the balloon of the Foley catheter that was passed leading to fistula formation.

Our patient presented with straining and passage of scanty urine which was subsequently followed by lack of volition to micturate. He also noticed passage of frequent watery stool. These modes of presentation veer from the usual symptoms, which include pneumaturia, faecaluria, and recurrent urinary tract infection among others, which are associated with VCF resulting from other causes [1–5]. It was suggested that the higher pressure in the colon may be responsible for those classical symptoms rather than the rectal micturation our patient presented with. As shown in the cystography and the intraoperative findings, the diameter of the fistulous connection was quite wide, 3.5 centimetres, as compared with common findings in fistulae from diverticulitis or malignant disease; this may explain the presentation of this patient.

Conventional radiological and urological techniques have been used for the confirmation of VCF with varying degree of sensitivity and specificity [9]. Barium enema was reported to have sensitivity of 20% to 62.5% in some series in addition to its importance in evaluating the underlying diseases. Some authors believed that computed tomography is the best diagnostic study with sensitivity of 40% to 100%. Cystoscopy, cystography, and magnetic resonance imaging are also useful. The diagnosis may still be difficult despite all these investigations. On few occasions surgical exploration may provide the final answer. To confirm the decision to perform surgical exploration others investigators have done the oral charcoal dye or Bourne tests while some even perform scintigraphy on the ingested radiographic tracers excreted in the urine. In the recent time oral ingestion of poppy seed has been used [10].

These tests are highly sensitive but they provide no information on the location or nature of a fistula. In our patient, the diagnosis was suspected clinically and only cystography was done to confirm the diagnosis. Aside from not being readily available, affordability may also constitute a financial constraint for the use of the aforementioned investigative modality in our setting.

It was believed that the width of the fistulous tract was wide enough for the cystography to demonstrate thus the need for further tests was not absolutely necessary.

Over the years the treatment options for VCF depends on the aetiological factor, clinical and investigation findings, and the general condition of the patient. Conservative measure with expectant closure of the fistula has been abandoned soon after the early description of this entity by West [11]. One form of surgical procedure or the other is generally necessary; with the advances in intensive care, antibiotics and nutritional supports the traditional three-stage operation has given way to a single stage operation without protective colostomy [12]. Our patient, in addition to the VCF, has malunion of left femoral facture and urethro- cutaneous fistula that definitely require surgical correction. Thus staging the repair of the VCF will add to the cost of his management. He had single stage repair of the fistula with protective cystostomy for the urethrocutaneous fistula which was also repaired at a later date. He did well with minimal morbidity of wound infection. He had since been discharged for orthopaedic consultation.

## 4. Conclusion

Urogenital injuries in association with injuries to other systems are not uncommon. Traumatic vesicoenteric fistula complicating missed blunt abdominal injury is an extremely rare occurrence in clinical practise. Timely identification, an in-depth clinical evaluation, and attention to details are of utmost importance in order to recognise and treat such injury to avoid the morbidity usually associated with it.
